# Minimally Invasive Distal Pancreatectomy as the Standard of Care in the US: Are We There Yet?

**DOI:** 10.3390/cancers17183015

**Published:** 2025-09-16

**Authors:** Laleh Foroutani, Andrew Gonzalez, Jaeyun Jane Wang, Bahaa I. Aburayya, Amir Ashraf Ganjouei, Jean Feng, Lucas Willian Thornblade, Kenzo Hirose, Ajay V. Maker, Eric Nakakura, Carlos Uriel Corvera, Adnan Alseidi, Mohamed A. Adam

**Affiliations:** 1 Department of Surgery, University of California San Francisco, San Francisco, CA 94143, USA; laleh.foroutani@ucsf.edu (L.F.); andrew.gonzalez@ucsf.edu (A.G.); jaeyunjane.wang@ucsf.edu (J.J.W.);; 2 Faculty of Medicine, Jordan University of Science and Technology, Irbid 22110, Jordan; 3 Department of Epidemiology and Biostatistics, University of California, San Francisco, CA 94158, USA

**Keywords:** distal pancreatectomy, joinpoint regression, minimally invasive surgery, laparoscopic distal pancreatectomy, robotic distal pancreatectomy, open distal pancreatectomy, surgical trends, postoperative pancreatic fistula

## Abstract

Introduction: While there is an increasing shift towards minimally invasive distal pancreatectomy (MIDP), little is known about how utilization of MIDP vs. open distal pancreatectomy (ODP) has evolved over time. We aimed to determine competing temporal trends in use and outcomes of MIDP (laparoscopic and robotic) over time and to determine if a threshold of effectiveness has been reached. Methods: Adults undergoing MIDP and ODP were identified from the National Cancer Database (2010–2021) and the National Surgical Quality Improvement Program database (2014–2022). Propensity score matching (PSM) was performed to address baseline differences between groups before comparing outcomes. Joinpoint regression analysis (JRA) was employed to assess adjusted trends in adoption and outcomes. We calculated Annual Percentage Change (APC) and Average Annual Percentage Change (AAPC) to quantify yearly adoption rates and their trends, respectively. Results: Among 21,966 patients in the NCDB cohort, 49.5% underwent MIDP, including 33.7% laparoscopic distal pancreatectomy (LDP) and 15.8% robotic distal pancreatectomy (RDP), while 50.5% underwent ODP. ODP declined from 74.1% of cases (2010) to 41.1% (2021), with an AAPC of −4.9%. MIDP increased significantly throughout the study from 25.9% of cases (2010) to 58.9% (2021), with an AAPC of 6.3%. Among MIDP subgroups, there was an initial increase in LDP until 2016, after which its rate of utilization stagnated with an AAPC of 0.7% (*p* > 0.05). In contrast, RDP demonstrated steady growth with an AAPC of about 15% (*p* < 0.05). A consistent and significant decline in clinically relevant postoperative pancreatic fistula rates occurred across all surgical approaches, with the most pronounced improvement observed in the robotic approach. MIDP approaches had significantly shorter hospital stays and lower mortality rates; however, RDP cases were associated with longer operative times compared to LDP and ODP. Conclusions: Over the past decade, the use of MIDP increased while ODP decreased. This increase was initially driven by greater use of LDP, which plateaued after 2016, and was further driven by the increased use of the robotic approach. Specifically, RDP demonstrated consistent growth, while LDP showed a decline around 2016. These findings highlight changing practice patterns, accompanied by improvements across all surgical approaches. This may provide insights for clinical training and resource allocation.

## Simple Summary

Pancreatectomy can be performed using an open approach or minimally invasive techniques, including laparoscopic and robotic surgery. Minimally invasive methods are associated with potential benefits such as reduced postoperative pain, shorter hospitalization, and faster recovery, yet their adoption patterns and comparative outcomes in the United States remain incompletely characterized. Using two large national databases, we examined temporal trends, practice patterns, and postoperative outcomes for each approach over more than a decade. We found a marked shift from open surgery toward minimally invasive techniques, driven primarily by a steady increase in robotic procedures. This transition coincided with shorter hospital stays, lower mortality, and reduced rates of major complications, particularly postoperative pancreatic fistula. These results highlight evolving surgical practice and may inform resource allocation and surgical training in pancreatic surgery.

## 1. Introduction

Open distal pancreatectomy (ODP) was traditionally the standard approach for distal pancreatectomy (DP), offering direct access, excellent visualization, and reliable oncologic control. However, advancements in minimally invasive distal pancreatectomy (MIDP) have led to the increasing adoption of laparoscopic distal pancreatectomy (LDP) and later robotic distal pancreatectomy (RDP). These MIDP techniques offer potential benefits, including reduced postoperative pain, shorter hospital stays, and faster recovery. Despite these advantages, the technical and financial barriers to implementing MIDP have historically limited its widespread adoption compared to other abdominal surgeries [1,2].

Recent level 1 evidence and guidelines support the adoption of MIDP (LDP and RDP) for benign and select malignant tumors [1,3]. However, uncertainty remains regarding the adoption patterns of MIDP [4,5]. Furthermore, published data may be limited by a reliance on linear models, which can overlook the dynamic nature of surgical adoption and restrict insights into how the use and performance of LDP and RDP have diverged over time. Thus, assessing dynamic trends in surgical adoption and outcomes is essential to understanding the evolution of surgical practice.

This study aimed to not only evaluate dynamic trends in the national adoption of ODP, LDP, and RDP over the past decade, but also to evaluate postoperative outcomes, including clinically relevant postoperative pancreatic fistula (CR-POPF), oncologic safety, and mortality.

## 2. Materials and Methods

### 2.1. Study Population and Data Sources

This study utilized two major national databases: the National Cancer Database (2010–2021) and the National Surgical Quality Improvement Program database (2014–2022) [6,7]. Adult patients who underwent DP were included. Those with incomplete demographic, clinical, or surgical approach data were excluded. Each cohort was analyzed separately and stratified by surgical approach (RDP, LDP, and ODP), which was categorized based on the initial operative plan.

### 2.2. NCDB

The NCDB is a comprehensive, nationwide, facility-based clinical registry jointly managed by the American College of Surgeons and the American Cancer Society, capturing approximately 70% of all newly diagnosed cancers in the United States. For this study, the NCDB cohort included patients who underwent DP between 1 January 2010, and 31 December 2021.

Demographic variables collected included age, sex, race (White, Black, or other), Charlson–Deyo comorbidity score, insurance status (Medicare, Medicaid/governmental, private, or uninsured), and income level, derived from ZIP codes linked to U.S. Census data. Additionally, information on facility types, classified as academic, comprehensive, integrated, and community, was collected. Facility locations were categorized into four broader regions. These four regions were created by combining the nine divisions defined by the U.S. Census Bureau: the Northeast (New England and Middle Atlantic), the Midwest (East North Central and West North Central), the South (South Atlantic, East South Central, and West South Central), and the West (Mountain and Pacific regions).

The surgical approach was determined based on the operative note at the start of the procedure. Indications for surgery included pancreatic ductal adenocarcinoma (PDAC), pancreatic neuroendocrine tumors (PNET), and other conditions. Diagnosis codes (ICD-O-3) for PDAC included 8140, 8210–8211, 8310, 8440, 8450–8453, 8470–8473, 8480, 8500–8503, 8507, 8560–8562, 8570–8572, and 8574–8576. PNET cases were classified under 8013, 8150–8156, and 8246. Additionally, tumor grade, stage, size, receipt of chemotherapy and radiotherapy, and evidence of lymphovascular invasion were assessed. Clinical outcomes included margin status (R0 for negative margins), the number of lymph nodes (LN) harvested, length of hospital stay (LOS) in days, 30-day readmission, 30-day and 90-day mortality rates, and 5-year survival.

Temporal trends in the adoption of ODP, LDP, and RDP were analyzed over the study period. This analysis compared all three approaches, assessing shifts in utilization over time. Additionally, we examined the trends of MIDP by comparing LDP and RDP against ODP. Patterns of accelerated uptake, plateau, and decline were identified, along with key inflection points marking significant shifts in surgical practice.

Finally, trends in clinical outcomes were assessed, including length of stay (LOS), achievement of negative surgical margins (R0), rates of conversion from MIS to open surgery, and five-year survival.

### 2.3. NSQIP

The NSQIP database is a multi-institutional dataset that collects perioperative data to evaluate surgical outcomes. The NSQIP cohort included patients who underwent DP from 1 January 2014, to 31 December 2022. Collected variables included demographic data such as age, sex, race, American Society of Anesthesiologists (ASA) classification, and indication for surgery. Operative data, including the type of surgical approach (ODP, LDP, RDP), operative time (in minutes), vascular resection, drain placement, and LOS (in days) were also recorded. CR-POPF was categorized into grade B or C using the International Study Group on Pancreatic Fistula (ISGPF) guidelines based on clinical severity [8]. Additionally, temporal trends in the rates of CR-POPF were analyzed by surgical approach over the study period.

### 2.4. Statistical Analysis

Descriptive statistics were used to summarize patient demographics and clinical characteristics. Continuous variables were reported as medians with interquartile ranges (IQRs), while categorical variables were presented as frequencies and percentages. For comparisons across the three surgical approaches (ODP, LDP, and RDP), the Kruskal–Wallis test was used for continuous variables and the Chi-square test was applied to categorical variables. A post hoc analysis compared LDP and RDP directly, using the Wilcoxon rank-sum test for continuous variables and pairwise Chi-square tests with Bonferroni correction for categorical variables.

A matched cohort analysis was performed using propensity score matching (PSM) via the *MatchIt* package (version 4.7.2) in R (version 4.5.0; R Foundation for Statistical Computing, Vienna, Austria) [9]. Patients were matched 1:1 across the three surgical approaches using nearest-neighbor matching based on age, tumor size, pathology, and stage. After matching, outcomes were reassessed and compared using appropriate statistical tests. These statistical analyses were performed using R software, with statistical significance set at *p* < 0.05.

Temporal trends in surgical approach adoption and clinical outcomes were analyzed using Joinpoint Regression Analysis (JRA). This method detects inflection points where a trend significantly changes direction by applying a piecewise log-linear model to identify distinct segments with varying rates of change over time. A one-year interval was used between joinpoints, with the optimal number of joinpoints determined using the Weighted Bayesian Information Criterion (WBIC).

Trends were analyzed by fitting a log-linear model, where the Annual Percentage Change (APC) was calculated for each segment to quantify the rate of change. The Average Annual Percentage Change (AAPC), a weighted summary of APCs across all segments, was also computed to provide an overall weighted measure of temporal trends [10]. The Joinpoint Regression Program (version 4.9.0.0; National Cancer Institute, Bethesda, MD, USA) was used, and statistical significance was tested using a Monte Carlo permutation method, with a two-sided significance threshold of *p* < 0.05 [11].

## 3. Results

### 3.1. NCDB Cohort Demographics and Clinical Characteristics

In the NCDB cohort, 21,966 patients underwent DP from 2010 to 2021, with 50.5% undergoing ODP, 33.7% LDP, and 15.8% RDP. There were no significant differences in patient age, sex, and Charleson-Deyo score across surgical cohorts. Patients who were White, lived in higher income bracket ZIP codes, and were privately insured were more likely to undergo MIDP (LDP or RDP) (*p* < 0.01). Academic centers were more likely to perform LDP, while comprehensive community centers were less likely to perform LDP. Integrated community centers were more likely to perform LDP and RDP. There were notable differences in regional distribution, with RDP being the most common in the Northeast, ODP in the South, and LDP in the West (*p* < 0.01) (Table 1).

Within the NCDB cohort, indication distributions showed that in the open group, PDAC accounted for 59.6% of the cases, followed by pNET 13.8%. In the laparoscopic group, PDAC accounted for 49.0% and pNET 13.4%. Among robotic cases, PDAC accounted for 44.1% and pNET 10.1%. MIDP approaches were more often employed for patients with favorable tumor grades and stages (*p* < 0.01). Patients with larger tumors and those who received radiotherapy or perioperative chemotherapy were more frequently managed with ODP (Table 1). We also evaluated the proportion of distal pancreatectomies performed without lymphadenectomy, defined as cases with zero lymph nodes examined. This occurred in 8.1% of open, 9.7% of laparoscopic, and 11.9% of robotic cases.

After propensity score matching, LOS was 5 days for MIDP and 6 days for ODP (*p* < 0.01). The rate of conversion to open surgery was highest in LDP. Notably, ODP demonstrated a significantly higher lymph node harvest compared with MIDP approaches. Both 30-day and 90-day mortality rates were highest for ODP (1.5% and 3.3%, respectively, *p* < 0.01) and lowest for RDP (0.6% and 1.7%, respectively, *p* < 0.01). 5-year survival was highest in patients who underwent RDP and ODP (*p* < 0.01). (Table 2).

### 3.2. Trends in the Use of Surgical Approaches and Outcomes

**Figure 1:**
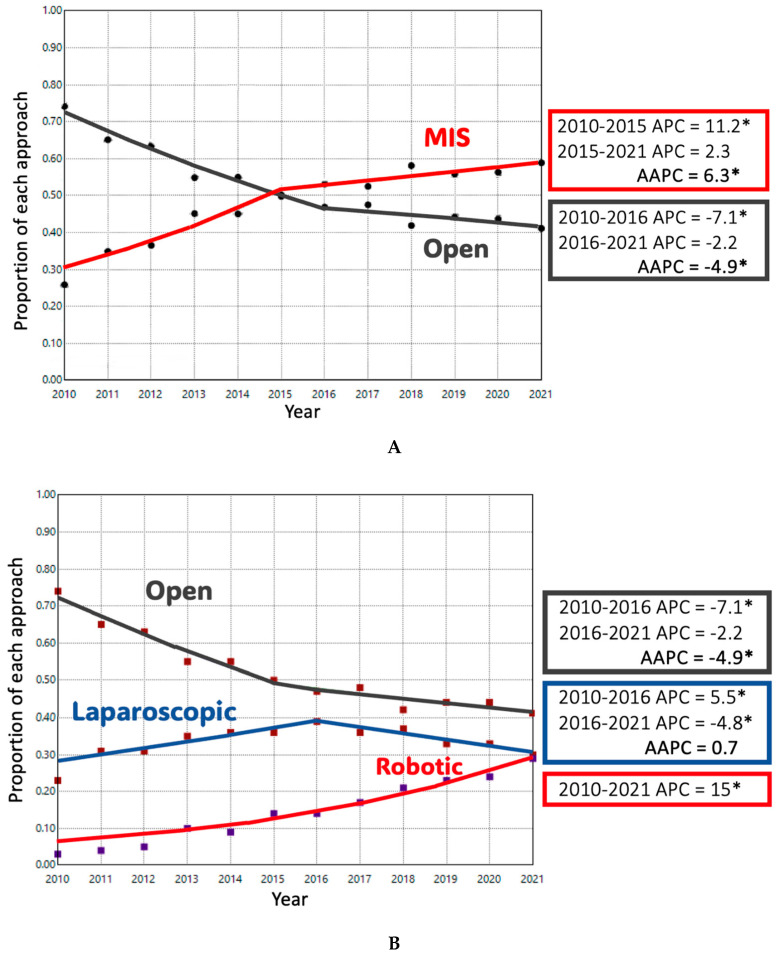
(**A**) Proportion of Open vs. Minimally Invasive Surgical (MIS) Approaches for Distal Pancreatectomy in the NCDB. APC: Annual Percent Change; AAPC: Average Annual Percent Change; *: *p*-value < 0.05. (**B**) Adjusted Trends of Open, Laparoscopic, and Robotic Distal Pancreatectomy Approaches in the NCDB.

**Figure 2:**
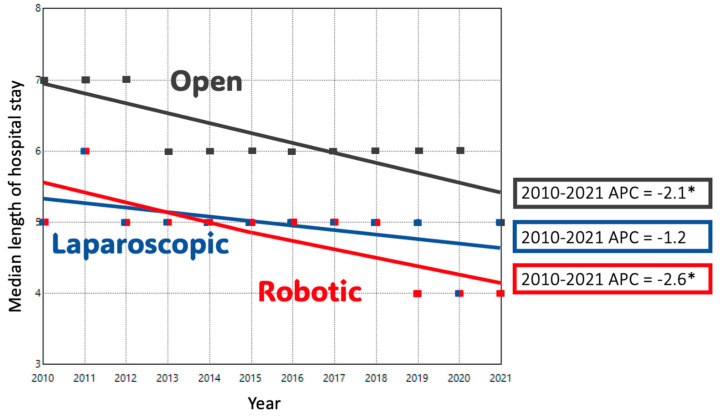
Trends in the Median Length of Hospital Stay in Distal Pancreatectomy by Surgical Approach; *: *p*-value < 0.05.

**Figure 3:**
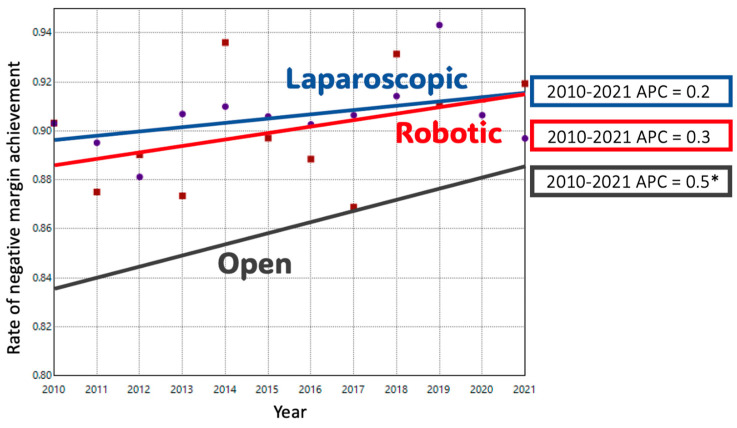
Trends in Negative Margin (R0) Achievement in Distal Pancreatectomy by Surgical Approach; *: *p*-value < 0.05.

**Figure 4:**
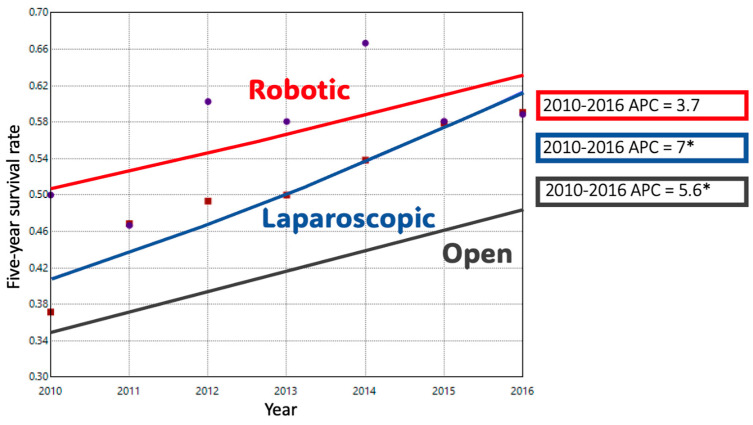
Trends in the Five-Year Survival Rate for Minimally Invasive Distal Pancreatectomy by Surgical Approach; *: *p*-value < 0.05.

**Figure 5:**
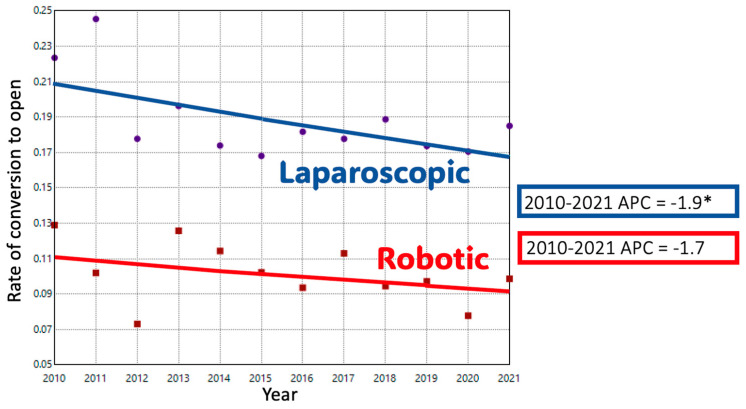
Trends in Conversion in Minimally Invasive Distal Pancreatectomy by Surgical Approach; *: *p*-value < 0.05.

ODP comprised 74.1% of cases in 2010; this decreased to 41.1% of cases by 2021. Conversely, MIDP represented 25.9% of cases in 2010 and 58.9% of cases in 2021. The adjusted trend analysis showed a significant decline in the use of ODP (AAPC-4.9%), with an inflection point in 2016 when the slope of decline decreased slightly (*p* < 0.05) (Figure 1A). MIDP had a period of significant growth until 2015, after which a slower and non-significant increase occurred until 2021 (AAPC 6.3%, *p* < 0.05). When MIDP was subdivided, LDP remained the dominant minimally invasive approach for much of the study period. Its utilization increased until 2016, after which it plateaued, resulting in a non-significant overall increase (AAPC + 0.7%). In contrast, RDP cases had a consistent, upward trend during the study period with an APC of + 15% (*p* < 0.05). (Figure 1B) Length of stay decreased significantly over time in both ODP and RDP groups. (Figure 2) The rate of R0 surgical margin increased across all groups during the study. (Figure 3) Five-year survival rates improved in both the ODP and MIDP groups. (Figure 4) LDP was the only group to experience a significant improvement in the rate of conversions during the study period (Figure 5).

### 3.3. NSQIP Cohort Demographics and Clinical Characteristics

We identified 18,667 patients who underwent DP in the NSQIP database between 2014 and 2022, with 49.1% undergoing ODP, 34.1% LDP, and 16.8% RDP. Female patients were more likely to undergo LDP and RDP (*p* < 0.01). White patients were more likely to undergo RDP (78.9%) (*p* < 0.01). Patients with higher ASA class were more likely to undergo ODP, while patients with lower ASA class (I and II) were more likely to undergo MIDP (*p* < 0.05). In the NSQIP cohort, which more reliably captured both benign and malignant indications, PDAC was the most frequent diagnosis, accounting for 35.6% of open cases, 43.7% of laparoscopic cases, and 42.2% of robotic cases. pNET was the second most common, accounting for 17.0% of open, 28.0% of laparoscopic, and 27.6% of robotic cases. Benign and cystic lesions were also well represented: pancreatitis accounted for 11.3% of open, 6.6% of laparoscopic, and 7.6% of robotic cases, while IPMN made up 7.4%, 11.2%, and 13.6%, respectively.

### 3.4. Trends in CR-POPF

**Figure 6:**
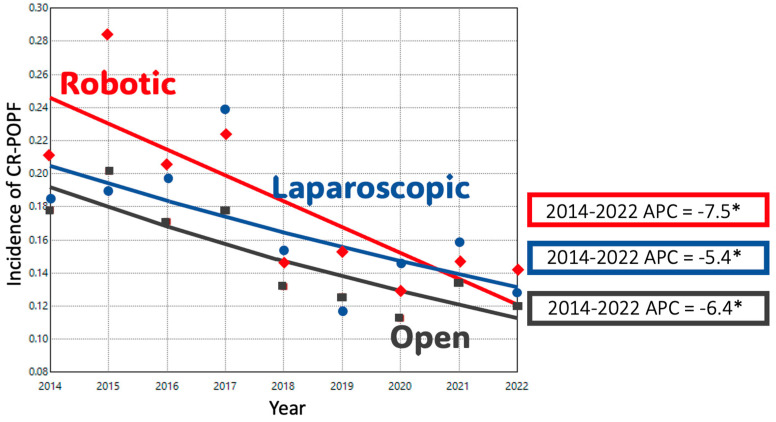
Trends in Clinically Relevant Postoperative Pancreatic Fistula (CR-POPF) Rates in Distal Pancreatectomy by Surgical Approach (NSQIP); *: *p*-value < 0.05.

The rates of CR-POPF varied significantly, with RDP having the highest rate, followed by LDP and ODP (*p* < 0.05). (Table 3) From 2014 to 2022, CR-POPF rates improved significantly across all surgical approaches. The initial rate of CR-POPF for ODP was 17.8%, which steadily declined to 12% by 2022 (APC = −6.4%). LDP had a higher initial CR-POPF rate of 18.5%, which significantly decreased to 12.8% by 2022 (APC = −5.4%). Lastly, RDP had the highest initial CR-POPF rate and exhibited the most pronounced decline (APC = −7.5%) (Figure 6).

## 4. Discussion

The aim of this study was to characterize dynamic trends in surgical approaches for DP over the past decade in the U.S. This contemporary study employed JRA to identify time periods associated with previously unrecognized shifts in the adoption rates of ODP and MIDP, highlighting the practical application of non-linear analytical approaches in examining surgical trends. There was a significant overall decrease in the utilization of ODP, with an annual adjusted decrease of 4.9%. This was accompanied by an uptrend in the utilization of MIDP approaches, which was initially driven by LDP and later by RDP. LDP initially showed an uptrend but plateaued after 2016, while RDP continued to increase. Our findings demonstrated improvements in CR-POPF across all approaches over time, most notably in RDP.

A major finding of this study was the notable decline in the rate of ODP utilization by the end of the study period in 2021. This was juxtaposed by a concurrent uptrend in MIDP, initially driven by LDP, while the subsequent rise was driven by the adaptation of RDP. This trend has been noted by prior studies. Jehan et al. examined 13,000 DP cases from the NCDB database and found that MIDP rates increased from one in every four patients in 2010 to one in every other patient in 2017 [12]. Hoehn et al. investigated robotic pancreatic surgery and noted significant increases in the adoption of RDP across three retrospective NCDB study periods between 2010 and 2016 [13]. Importantly, our study offers an up-to-date analysis supported by a robust sample size from two national databases.

Prior studies evaluating trends in pancreatic surgery have often relied on linear models, which assume a constant rate of change and fail to capture dynamic shifts in practice patterns [14,15]. A key strength of this study is the use of JRA methodology, which identified time points in both MIDP and ODP that reflect reductions in the rates of annual change. To our knowledge, these inflection points have not been described in previous studies, likely due to the limitations of traditional, linear analyses. Furthermore, using JRA, we were able to detect more nuanced patterns in the data. For example, the overall AAPC of LDP adoption rates was not significant despite an initially significant increase in utilization, due to a plateau in use after 2016. This finding challenges the notion that LDP utilization lagged throughout the entirety of the past decade [12]. In addition, many prior studies do not differentiate between LDP and RDP when describing the increase in utilization of MIDP, thus failing to capture divergent adoption trajectories [13]. Our study is the first to demonstrate that RDP began to replace LDP as the dominant MIDP approach in 2016, which coincides with a surge in research efforts showing comparable outcomes between RDP and LDP [16,17].

Our analysis demonstrated that MIDP overtook ODP as the predominant approach by 2015. The initial growth of MIDP was largely driven by LDP, which plateaued after 2016, coinciding with a reduction in annual adoption rates. Surgeons initially trained in LDP may have continued to favor this approach, resulting in a stable population of surgeons performing LDP. Subsequent growth in MIDP was later attributable to RDP. This transition may reflect a generational shift in training, increased comfort with robotic technology, and expansion of robotic programs, especially following the widespread dissemination of the da Vinci Xi system in 2014, which facilitated greater institutional adoption [18,19]. However, it is important to note that increased access to robotic platforms does not necessarily translate into their routine use, as adoption depends on multiple factors, including surgeon experience, institutional resources, and patient selection.

While national studies have supported an overall increase in MIDP, few have examined geographic disparities within the U.S. This study noted both regional and institutional variations. LDP was more common on the West Coast, whereas RDP was more common in the Northeast. Academic centers showed higher adoption of LDP and RDP, whereas community hospitals predominantly performed ODP, suggesting disparities in access to robotic platforms. Institutions with fewer resources face challenges acquiring and maintaining robotic systems due to the high costs involved [12]. The steep learning curve, credentialing requirements, and higher costs of RDP may also limit broader adoption in resource-poor areas [12,20]. We also found that patients who were White, lived in higher income bracket ZIP codes, and were privately insured were more likely to undergo MIDP. Such disparities in MIDP access are well documented, with Black, Hispanic, and uninsured patients being significantly less likely to undergo MIDP [12].

The trends in CR-POPF rates are particularly noteworthy, as CR-POPF remains a major complication following DP. Our study demonstrates a consistent decline in CR-POPF rates across all surgical approaches, including ODP. These improvements are likely due to the parallel implementation of Enhanced Recovery After Surgery (ERAS) protocols, better patient selection, and improvements in surgical techniques that all enhanced outcomes. Boyev et al. reported a 24% decline in CR-POPF rates across approaches during a six-year period [21]. Technical advancements, including improved stapling technology, refined suturing techniques, and the use of adjuncts such as omental flaps, may also have contributed to these improvements [22,23,24]. Overall, CR-POPF rates were similar for minimally invasive approaches, but RDP showed more pronounced improvement. Early in the study period, higher CR-POPF rates may reflect the learning curve, while subsequent declines may be explained by increased surgeon experience and the potential technical advantages of RDP, such as more meticulous dissection.

Improvements were also seen in other perioperative metrics after MIDP, including R0 margin status, LOS, and mortality. Molina et al. also reported improved outcomes such as decreased operative time, estimated blood loss, and length of stay in RDP [25,26]. Thus, our study contributes to a growing body of literature supporting safe outcomes associated with MIDP compared to ODP [1,27]. It is important to note, however, that demonstrating comparable safety or outcomes does not imply that one specific technique should be universally adopted. As surgeons progress along the learning curve, operative times tend to decrease while technical precision improves, contributing to the observed reduction in complications for a given approach [2,20].

While the aim of this study is not to provide health policy recommendations, these findings should be cautiously interpreted within the context of value-based care. Our data and prior studies suggest that MIDP, including RDP, can offer improvements in perioperative outcomes such as LOS, mortality, and R0 margin status. However, broader adoption of RDP must also be weighed against its substantial purchase, maintenance, and training costs, as well as longer operative times compared with LDP. Accordingly, it is important to acknowledge that a purposeful effort should direct the resources to policies with greater population-level benefit rather than those with limited incremental value. This underscores the need for future research that integrates both clinical and economic outcomes to guide sustainable adoption. This study has potential limitations inherent to large-scale database research, including the inability to infer causation from retrospective data and the introduction of confounding variables. PSM mitigates some of these concerns but cannot account for all potential confounders. The NCDB does not capture benign indications, limiting the scope of comparison, whereas NSQIP provides more detailed categorization. In addition, both databases harbor missing or incomplete data and lack granularity for some variables, such as operative details (e.g., spleen preservation), detailed operative indications, tumor biology, and surgeon volume, which may introduce selection bias.

Our study has important implications. It provides insights that could inform policy, credentialing, and resource allocation.

Future studies should examine how hospital volume, surgeon specialization, and access to robotic technology influence regional and institutional disparities in MIDP access. Further research should also evaluate how operative efficiency evolves with surgeon experience and whether workflow standardization (e.g., port placement, instrument handling) can improve outcomes [3,28]. Lastly, efforts to minimize barriers for patients most likely to benefit from each approach, particularly those with complex pathology or high-risk comorbidities, may enhance equitable access to advanced surgical care across socioeconomic groups [29].

## 5. Conclusions

Our findings demonstrate the increasingly important role that MIDP plays in distal pancreatectomy. This shift is accompanied by improved outcomes, highlighting the growing potential for the safe implementation of robotic surgery for the management of pancreatic pathologies.

## Figures and Tables

**Table 1:** Demographic and Clinical Characteristics of Patients Undergoing Distal Pancreatectomy in the NCDB.

Variable	Robotic *n* = 3464	Laparoscopic *n* = 7406	Open *n* = 11,096	All Patients *n* = 21,966	*p*-Value	*p*-Value (Lap vs. Rob)
Age, years (median, IQR)	65 (56, 73)	65 (56, 73)	65 (56, 73)	65 (56, 73)	0.8	0.53
Sex Female	49%	50%	51%	50%	0.12	0.5
Race					0.01 *	0.67
White	84%	84%	83%	83%		
Black	11%	11%	12%	12%		
Other	5%	5%	5%	5%		
Charlson–Deyo Score					0.2	0.27
0	62%	64%	63%	63%		
1	24%	24%	25%	25%		
2	8%	7%	7%	7%		
3	6%	5%	5%	5%		
Income					<0.01 *	0.16
< $30,000	14%	13%	16%	15%		
$30,000–$34,999	18%	20%	20%	20%		
$35,000–$45,999	25%	24%	24%	24%		
≥ $46,000	43%	43%	40%	41%		
Insurance					<0.01 *	0.65
Medicare	51%	50%	50%	50%		
Private Insurance	40%	41%	39%	40%		
Medicaid/governmental	7%	7%	8%	7%		
Not Insured	1%	1%	2%	2%		
Facility type					<0.01 *	<0.01 *
Academic	55%	59%	56%	57%		
Comprehensive	24%	19%	24%	23%		
Integrated	20%	20%	18%	19%		
Community	1%	1%	2%	1%		
Facility location					<0.01 *	<0.01 *
South	33%	33%	36%	34%		
Midwest	22%	23%	24%	24%		
Northeast	28%	23%	22%	23%		
West	11%	16%	13%	14%		
Indication					<0.01 *	<0.01 *
PDAC	44%	49%	60%	54%		
PNET	10%	13%	14%	13%		
Other	46%	38%	27%	33%		
Grade					<0.01 *	<0.01 *
I	45%	42%	33%	37%		
II	28%	30%	33%	32%		
III	10%	14%	17%	15%		
IV	1%	1%	1%	1%		
Stage					<0.01 *	<0.01 *
I	45%	43%	35%	39%		
II	39%	42%	45%	43%		
III	7%	5%	6%	6%		
IV	3%	4%	8%	6%		
Tumor size, cm (median, IQR)	2.6 (1.7, 4)	2.9 (1.8, 4.2)	3.3 (2.1, 5)	3 (2, 4.5)	<0.01 *	<0.01 *
Chemotherapy	31%	35%	45%	40%	<0.01 *	<0.01 *
Radiotherapy	6%	9%	14%	11%	<0.01 *	<0.01 *
Lymph vascular invasion	31%	32%	36%	34%	<0.01 *	0.47
Number of LN harvested (median, IQR)	11 (4, 18)	11 (5, 18)	12 (5, 18)	11 (5, 18)	<0.01 *	0.42
Negative surgical margin	90.6%	90.8%	86%	88%	<0.01 *	0.85
Length of Stay, days (median, IQR)	5 (4, 6)	5 (4, 7)	6 (5, 8)	5 (4, 7)	<0.01 *	<0.01 *
Re-admission	8.5%	7.6%	8.5%	8.2%	0.08	0.14
30-day mortality	0.6%	1.1%	1.9%	1.4%	<0.01 *	<0.01 *
90-day mortality	1.6%	2.2%	4.2%	3.1%	<0.01 *	<0.01 *
Five-year survival	46.2%	45.5%	35.8%	40.2%	<0.01 *	<0.01 *

*: Indicates statistical significance (*p*-value < 0.05); Lap: Laparoscopic approach; Rob: Robotic approach; PDAC: Pancreatic Ductal Adenocarcinoma; PNET: Pancreatic Neuroendocrine Tumor; LN: Lymph nodes.

**Table 2:** Clinical Characteristics of Patients Undergoing Distal Pancreatectomy in the NCDB After matching for Age, Histology, Stage, and Tumor size.

Variable	Robotic *n* = 3382	Laparoscopic *n* = 3382	Open *n* = 3382	All Patients *n* = 10,146	*p*-Value	*p*-Value (Lap vs. Rob)
Number of LN harvested (median, IQR)	11 (4, 18)	11 (5, 18)	12 (5, 18)	11 (5, 18)	<0.01 *	0.42
Negative surgical margin	90.7%	89.1%	89.7%	89.8%	<0.01 *	<0.01 *
Length of Stay, days (median, IQR)	5 (4, 6)	5 (4, 7)	6 (5, 8)	5 (4, 7)	<0.01 *	<0.01 *
Re-admission	8.3%	7.8%	8.6%	8.2%	<0.01 *	0.02 *
30-day mortality	0.6%	1.3%	1.5%	1.1%	<0.01 *	<0.01 *
90-day mortality	1.7%	2.4%	3.3%	2.5%	<0.01 *	<0.01 *
Five-year survival	46.0%	38.1%	46.4%	43.0%	<0.01 *	<0.01 *

*: Indicates statistical significance (*p*-value < 0.05).

**Table 3:** Demographic, Clinical, and Perioperative Characteristics of Patients Undergoing Distal Pancreatectomy in the NSQIP.

Variable	Robotic *n* = 3140	Laparoscopic *n* = 6356	Open *n* = 9171	All Patients *n* = 18,667	*p*-Value	*p*-Value (Lap vs. Rob)
Age, years (median, IQR)	64 (57, 75)	64 (56, 75)	64 (57, 75)	64 (57, 75)	1	1
Sex Female	56%	56.1%	53%	54.6%	<0.01 *	0.87
Race					<0.01 *	<0.01 *
White	78.9%	67.5%	72.5%	71.9%		
Black	9.6%	8.5%	9.7%	9.3%		
Other	5.3%	6.3%	4.6%	5.3%		
ASA					<0.01 *	<0.01 *
I	1.2%	1.4%	0.8%	1.1%		
II	28.7%	30.5%	23.1%	26.6%		
III	66.4%	62.6%	69%	66.4%		
IV	3.8%	5.4%	6.8%	5.8%		
V	0%	0%	0.2%	0.1%		
Indication					<0.01 *	<0.01 *
PDAC	42.2%	43.7%	35.6%	29.5%		
PNET	27.6%	28%	17%	22.5%		
Pancreatitis	7.6%	6.6%	11.3%	9.1%		
IPMN	13.6%	11.2%	7.4%	9.7%		
Vascular resection					<0.01 *	<0.01 *
No	95.8%	93.8%	87.9%	91.2%		
Artery	1.2%	0.8%	2.1%	1.5%		
Vein	1.3%	1.9%	5.7%	3.6%		
Both	1.4%	2.6%	2.8%	2.5%		
Drain Placement	90.7%	86.6%	84.9%	86.5%	<0.01 *	<0.01 *
Operative time, minutes (median, IQR)	246 (191, 319)	204 (155, 267)	217 (156, 298)	217 (161, 291)	<0.01 *	<0.01 *
Length of Stay, days (median, IQR)	5 (4, 6)	5 (4, 6)	6 (5, 8)	5 (4, 7)	<0.01 *	1
CR-POPF	17.10%	16.88%	14.91%	15.95%	<0.05 *	0.82

*: Indicates statistical significance (*p*-value < 0.05); CR-POPF: Clinically Relevant Postoperative Pancreatic Fistula.

## Data Availability

Restrictions apply to the availability of these data. Data were obtained from the National Cancer Database (NCDB) and the American College of Surgeons National Surgical Quality Improvement Program (ACS NSQIP). Access to these datasets is subject to approval and data-use agreements with the American College of Surgeons. Further details are available at: NCDB: https://www.facs.org/quality-programs/cancer-programs/national-cancer-database (accessed on 11 September 2025). ACS NSQIP: https://www.facs.org/quality-programs/data-and-registries/acs-nsqip/participant-use-data-file (accessed on 11 September 2025).

## Abbreviations

The following abbreviations are used in this manuscript:

MIDP	Minimally invasive distal pancreatectomy
LDP	Laparoscopic distal pancreatectomy
RDP	Robotic distal pancreatectomy
ODP	Open distal pancreatectomy
CR-POPF	Clinically relevant postoperative pancreatic fistula
NCDB	National Cancer Database
NSQIP	National Surgical Quality Improvement Program
APC	Annual percent change
AAPC	Average annual percent change
PSM	Propensity score matching
LOS	Length of stay
ISGPF	International Study Group on Pancreatic Fistula
PDAC	Pancreatic ductal adenocarcinoma
PNET	Pancreatic neuroendocrine tumor
